# X-ray fluoroscopy guided localization and steering of miniature robots using virtual reality enhancement

**DOI:** 10.3389/frobt.2024.1495445

**Published:** 2024-11-13

**Authors:** Husnu Halid Alabay, Tuan-Anh Le, Hakan Ceylan

**Affiliations:** ^1^ Department of Physiology and Biomedical Engineering, Mayo Clinic College of Medicine and Science, Scottsdale, AZ, United States; ^2^ Max Planck Queensland Centre, Queensland University of Technology, Brisbane, QLD, Australia

**Keywords:** microrobot, millirobot, medical robot, digital twin, virtual reality, magnetic navigation, X-ray fluoroscopy

## Abstract

In developing medical interventions using untethered milli- and microrobots, ensuring safety and effectiveness relies on robust methods for real-time robot detection, tracking, and precise localization within the body. The inherent non-transparency of human tissues significantly challenges these efforts, as traditional imaging systems like fluoroscopy often lack crucial anatomical details, potentially compromising intervention safety and efficacy. To address this technological gap, in this study, we build a virtual reality environment housing an exact digital replica (digital twin) of the operational workspace and a robot avatar. We synchronize the virtual and real workspaces and continuously send the robot position data derived from the image stream into the digital twin with short average delay time around 20–25 ms. This allows the operator to steer the robot by tracking its avatar within the digital twin with near real-time temporal resolution. We demonstrate the feasibility of this approach with millirobots steered in confined phantoms. Our concept demonstration herein can pave the way for not only improved procedural safety by complementing fluoroscopic guidance with virtual reality enhancement, but also provides a platform for incorporating various additional real-time derivative data, e.g., instantaneous robot velocity, intraoperative physiological data obtained from the patient, e.g., blood flow rate, and pre-operative physical simulation models, e.g., periodic body motions, to further refine robot control capacity.

## Introduction

Milli- and microrobots have emerged as miniature mobile devices for conducting precise tasks within confined and hard-to-access spaces (Nelson et al., 2010; Sitti et al., 2015; Dupont et al., 2021). These devices can be operated with remote human-in-the-loop control or semi-autonomously by leveraging locally responsive designs to perform complex medical procedures once considered not possible or feasible (Ceylan et al., 2017; Ceylan et al., 2019a). The potential for these robots to impact areas such as targeted drug (Ceylan et al., 2019b) and cell delivery (Yasa et al., 2019; Dogan et al., 2022; Go et al., 2023), embolization (Liu et al., 2024), and clot removal (Khalil et al., 2018) has been increasingly demonstrated in various studies. Most of these applications have been realized using *in vitro* settings, and the localization and navigation tasks of miniature robots were accomplished under camera or optical microscopy.

The application of these robots in animal disease models or human patients introduces challenges due to the non-transparency of body tissues to visible and near-infrared light. Optical methods, such as fluorescence imaging and coherent optical tomography offer limited penetration, confining their use to superficial tissues (Bergeles et al., 2012). Consequently, there has been a shift towards utilizing medical imaging modalities (Ceylan et al., 2019a; Liu et al., 2024; Pané et al., 2018; Aziz et al., 2020; Iacovacci et al., 2019a).

Among these modalities, magnetic resonance imaging (MRI) stands out for its deep tissue penetration and high spatial resolution. Real-time MRI systems can potentially track miniature robots while offering localization capacity in plane resolution of around 2 mm (Li et al., 2024; Nayak et al., 2022). On the downside, MRI can introduce significant image distortions for magnetic miniature robots, which can be more than 10–50 times of the original robot size, potentially complicating fast and accurate localization and steering within confined and sensitive tissues (Shapiro et al., 2004; Tiryaki et al., 2022). Although certain dedicated robot designs have been steered using an MRI system (Li et al., 2024), many untethered miniature robots would face compatibility challenges with MRI’s strong magnetic fields. High purchase and operating costs of MRIs place an additional prohibitive barrier in developing new miniature robots, detection, localization, tracking steering.

Clinical ultrasonography provides (10–15 MHz) fast-imaging speed, safety, and cost-effectiveness, but it has lower spatial resolution and limited tissue penetration (3–4 cm) (Kremkau, 2015; Wu et al., 2019; Del Campo Fonseca et al., 2023). Lower frequencies (2–5 MHz) offer deeper penetration (up to 30 cm), which, however, comes at the cost of additionally reduced resolution (Ng and Swanevelder, 2011). Further, lower frequencies are more susceptible to scattering from bones and air pocket, which can degrade image quality for the robot localization and precise steering (Hausman et al., 2022).

X-ray fluoroscopy and positron emission tomography (PET), provide deep tissue penetration, high spatial resolution and near real-time acquisition, making them highly effective for the robot detection and robot tracking (Vilela et al., 2018; Iacovacci et al., 2019b; Ligtenberg et al., 2024). Miniature robots as small as 100 μm feature size can be detected using these imaging systems. However, these methods are not suitable for providing anatomical context around the robot workspace, which is crucial for precise localization and safe robotic navigation.

Hybrid image systems, such as PET/MRI and SPECT/CT, can overlay background anatomy with the imaging target (Voss, 2023). However, obtaining, maintaining and modifying such compact clinical systems for miniature robot development within research labs comes with insurmountable cost and technical challenges.

Additional detection and tracking experiments for miniature robots have utilized various non-clinical imaging systems, including closed-box X-ray imaging systems (Go et al., 2023; Wang et al., 2022), photoacoustic imaging (Wu et al., 2019) and magnetic particle imaging (MPI) (Bakenecker et al., 2021). Closed-box X-ray imaging systems, typically used in research labs, have notable drawbacks, such as the potential for providing misleading assessment about the robot visibility when transitioning to clinical settings with C-arms. C-arms are designed to minimize X-ray exposure using automatic exposure control algorithms and pulsed X-ray delivery. In contrast, closed-box systems often result in higher and continuous radiation exposure. Photoacoustic imaging is gaining traction in life sciences and pre-clinical studies for its ability to provide high-speed full-body imaging of small animals *in vivo* (Lin et al., 2021). Optical excitation provides rich contrast and detailed anatomy for the imaging of vascular structures, which could be very useful in endovascular interventions. However, penetration depth, unclear spatial accuracy and imaging targets other than superficial blood vessels in humans remains a challenge of this imaging technology (Steinberg et al., 2019). MPI is noted for its higher sensitivity and improved temporal resolution, which are advantageous for the detection of miniature robots. However, it falls short in providing anatomical context (Yang et al., 2022).

Given these limitations, there is an unmet need for integrated and accessible solutions capable of providing fast image acquisition, deep tissue penetration, and the capture of detailed anatomic features for the real-time tracking, precise localization, and steering of miniature robots. These limitations present a significant safety barrier to the translational development of miniature robots, particularly for high-stakes applications in areas such as neuro and endovascular interventions. To overcome these hurdles, it is essential to develop innovative concepts that advance the field towards a higher level of technological readiness level for innovative clinical applications (Ceylan et al., 2019a).

Virtual reality environments are increasingly recognized for their potential in medical education, diagnostics, and intervention planning (Sun et al., 2023). It offers enhanced visual feedback and user intuitiveness, crucial for the precise manipulation of miniature robots in complex medical scenarios. A very recent study reported an immersive magnetic manipulation of micro- and nanoparticles based on RGB camera image feedback, providing unobstructed 3D visualization and depth perception from a first-person perspective (Chowdhury et al., 2024). It allows users to dynamically shift viewpoints, enabling complete 360° visualization, thus eliminating the need to abstract data from static two-dimensional images. The ability to maneuver, scale, and rotate virtual reality perspectives dynamically enhances internal spatial representation and improves intuitiveness, potentially leveraging the safety for manipulating untethered miniature robots.

In this study, we address the challenges of miniature robot tracking, localization, and steering under fluoroscopic guidance by developing an innovative approach that incorporates a virtual reality environment. This virtual reality environment features a digital twin of the robot’s operational workspace and the miniature robot avatar. By synchronizing the coordinate systems of the virtual and real workspaces and continuously sending robot’s key identifier data processed from the clinical C-arm image stream, we update the robot avatar localized within the digital twin. Using this virtual reality feedback, we can robustly track and steer the robot with very short delay times without having to see the actual robot workspace.

We validate this concept by demonstrating the tracking and steering of magnetic millirobots within a branched luminal phantom and effectively navigating a maze. The virtual reality enhancement strategy is versatile and adaptable to other medical imaging modalities, including PET or ultrasonography. Additionally, this approach can be applied across various robot designs, provided they are compatible with the chosen imaging system and potentially to diverse biological environments such as *ex vivo* tissues, animals and human patient settings.

## Results

### Magnetic navigation under X-ray fluoroscopy guidance

**FIGURE 1:**
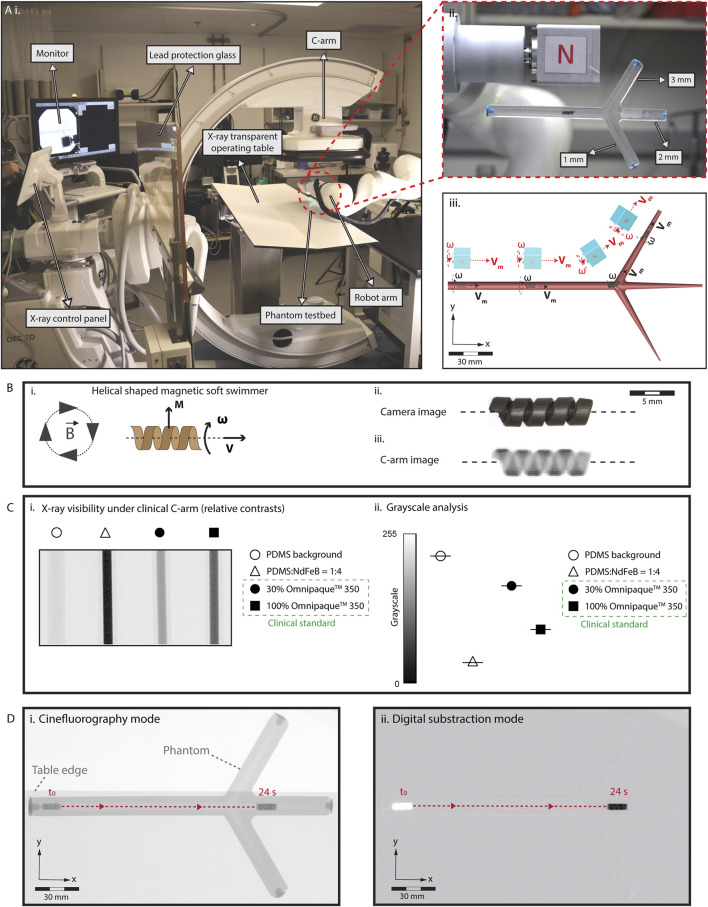
Limitations in the real-time localization and navigation of miniature robots under real-time X-ray fluoroscopy guidance. **(A)** (i) Our eX-MMPt system. (ii) For the remote magnetic manipulation, a permanent magnet is connected to a DC motor to create rotating magnetic fields. A two-dimensional phantom model is designed with branches to demonstrate precise localization and steering. (iii) The rotating motor is attached to the final joint of a seven-degree-of-freedom robot arm, allowing precise control over the direction and magnitude of the magnetic fields in the local three-dimensional workspace around the miniature robot from a mechanically safe position. **(B)** (i) A model helical-shaped miniature swimmer (HMS) that moves under rotating magnetic fields with cork-screw mode of locomotion. (ii), (iii) An RGB camera and X-ray fluoroscopy images of HMS. **(C)** (i) Relative X-ray contrast of HMS precursor composite inside 1 mL syringe with respect to Omnipaque™ 350 filled syringes. 30% Omnipaque™ 350 sets the lower boundary of iodine concentration that offer visibility within the human body. (ii) Using C-arm greyscale analysis of the relative X-ray contrasts showing HMS is reliably visible for real-time C-arm guided medical interventions inside the human body, as its relative x-ray contrast density surpass the clinical benchmark. **(D)** Visualization of HMS in the (i) cinefluorography and (ii) digital subtraction modes as it moves inside a fluid-filled phantom channel, demonstrating the poor anatomical detail of the environment in which the robot is navigated.

We built an X-ray fluoroscopy-guided magnetic manipulation platform (eX-MMPt) for remote navigation control of miniature robots, particularly in large animals and humans, as shown in Figure 1A. It utilizes a clinical C-arm to capture fluoroscopic videos in cinefluoroscopic (cine) (15 fps image acquisition rate) and digital subtraction (DS) (8 fps image acquisition rate) modes without significant distortion or loss of information. This is crucial for guiding the magnetic manipulation of miniature robots in near real time. Also, the open workspace of C-arm allows the successful operation of a magnetic manipulation platform without compromising the imaging quality or requiring substantial re-design of two parallel working systems. eX-MMPt involves a dipole permanent magnet mounted on the rotor of a DC motor, which can generate rotating magnetic fields up to 100 Hz. The motor is integrated to the final joint of a seven-degree-of-freedom robot arm, allowing precise control over the direction and magnitude of the magnetic fields from a mechanically safe position. This configuration allows a computer-programmable control of the local magnetic field magnitude, magnetic field gradient, direction, and rotation frequency around a magnetic miniature robot in a large volume that can accommodate a human subject.

For the robotic localization and steering demonstrations, we designed a magnetic, helical-shaped miniature swimmer (HMS) (Figure 1B). Analogous to our previous helical-shaped microswimmers, HMS is magnetized in the direction proportional to its major helical axis for -torque-dominant cork-screw locomotion (Lee et al., 2021; Hu et al., 2021). Detailed information regarding the fabrication and magnetization of HMS is provided in Supplementary Figure S1 and the Materials and Methods section. We placed HMS in a confined, fluid-filled two-dimensional luminal phantom with two branches to demonstrate the robot detection, localization, and steering capabilities.

Figure 1B illustrates the visibility of the HMS under C-arm. To ensure compatibility with clinical C-arms, it is imperative that the HMS provides sufficient contrast to maintain reliable visibility during procedures. Enhanced contrast enables better differentiation of the HMS from the surrounding tissues, which is crucial for accurate navigation. Additionally, the level of contrast affects the total exposure dose set by the automatic exposure control algorithms of the medical C-arms, which, subsequently influences the overall radiation dose received by the patient. To semi-quantitatively evaluate the X-ray contrast of the HMS, we compared its polymer precursor to iohexol, using its intravascular formulation, Omnipaque® 350, as a clinical benchmark. For real-time X-ray fluoroscopy-guided interventions, medical devices within the human body provide contrast levels at least equivalent to 30% Omnipaque® 350 (Zhang et al., 2022).

The greyscale analysis suggested that the relative contrast of the HMS polymer composition surpasses that of 100% Omnipaque®350, ensuring that the HMS is sufficiently visible for clinical applications (Figure 1C). This superior contrast is attributable to two primary factors: (i) the inclusion of NdFeB magnetic particles, averaging about 5 µm in size, within the HMS structure, which are inherently denser than iodine-containing molecules used in contrast agents, and (ii) the higher atomic number of neodymium (_60_Nd) compared to iodine (_53_I), which leads to better visibility under C-arm fluoroscopy due to increased X-ray attenuation.

To assess the visibility of HMS based on the robot size, we fabricated models ranging from 1.2 to 3.6 mm in diameter (Supplementary Figure S2A). X-ray contrast levels were not affected as the size decreased and the helical robot shapes were clearly distinguishable. However, due to the spatial resolution limitations of X-ray fluoroscopy, the finer details of the helix became less distinct with smaller sizes to the human eye.

When we applied magnetic actuation to the HMS within the phantom, the robot structure and locomotion were effectively captured using cine and DS imaging modes (Figure 1D, Supplementary Movie S1). In DS mode, a noticeable delay occurred before the robot moved from its initial position and became visible. This delay is attributable to the C-arm computer’s continuous digital subtraction, which removes each frame’s image from the baseline image to enhance target contrast. As HMS began to move within the channel, it visually emerged from the otherwise blank background, leaving behind a white digital mark at its starting location. Notably, in both imaging modes, the lumen and the remainder of the phantom were nearly invisible to the external operator. Additionally, the background visibility was compromised by significant fluctuations caused by the presence of high-contrast materials, such as the robot arm and a large permanent magnet, which entered the imaging field during the robot control. Lack of clear visibility in the structural details of the robot’s workspace and the and fluctuations of the robot visibility poses a risk the external operator’s ability to adjust the magnetic field strength and direction, which is critical for effective steering of the HMS.

### Creation of the virtual reality environment

**FIGURE 2:**
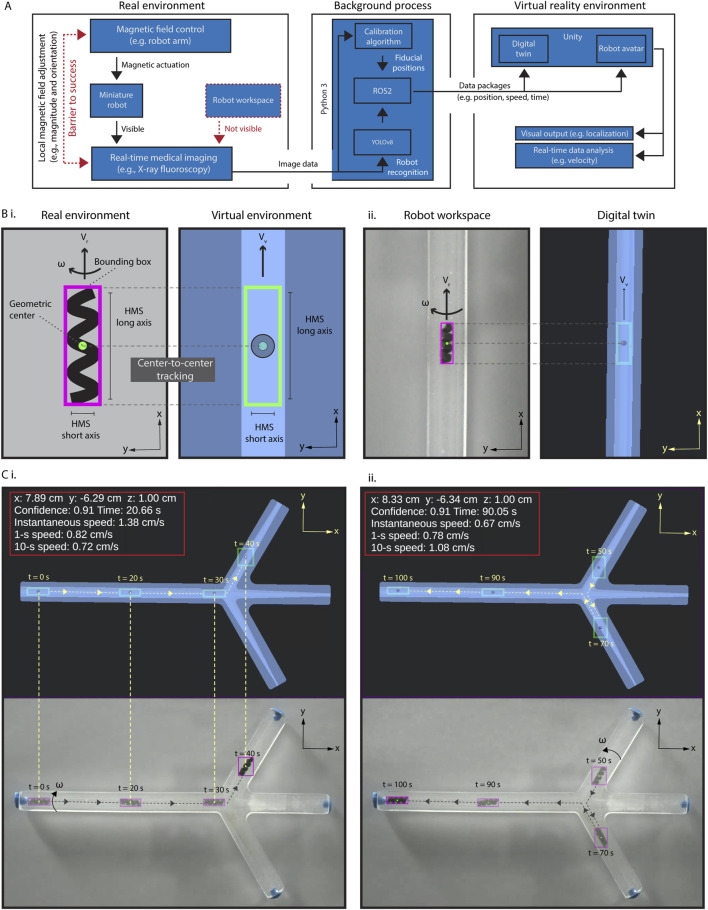
Creation and validation of the virtual interface. **(A)** The schematic diagram illustrating the data process and interface connections in the proposed virtual enhancement strategy. **(B)** (i) An illustration of our approach for HMS detection by the detection algorithm, with a bounding box created around it. A replicate of the bounding box, HMS avatar, is created as the digital twin of the HMS in Unity®, and the position of its geometric center is used to localize it in the digital twin of the phantom. (ii) Demonstrating synchronized detection and localization of HMS in real and virtual reality environments. **(C)** Validating the accurate and uninterrupted synchronization of an HMS avatar in the spatial and temporal domains using an RGB camera captured video, demonstrated with time stamps, position, and HMS speed data (i) First half of the movement from left to right (0s–40s), (ii) second half of the movement from right to left (50s–100s).

Figure 2A illustrates our data processing and connection flow from the real environments, i.e., actual position data derived from the C-arm stream to the virtual reality environment. To enhance tracking accuracy, we first developed a highly selective and sensitive object detection algorithm tailored for HMS (See Materials and Methods section for details). This training-based algorithm captures critical data about HMS, including the confidence value of the detection success rate, a bounding box surrounding the HMS body, the geometric center of the HMS, and the position data of the geometric center within the image frame (Figure 2B). The data is then sent to the digital twin to create a virtual avatar of HMS with matching identifiers, allowing for precise localization.

Our detection algorithm can successfully identify the HMS across sizes ranging from 1.2 to 3.6 mm in diameter, despite the difficulty of discerning helical features visually as the robot size diminishes (Supplementary Figure S2A). To rigorously test the object detection against contrast fluctuations where human eye may fail, we introduced an extreme X-ray contrast structure, a cylindrical N52 NdFeB magnet measuring 5.1 cm in length and diameter, into the workspace. This addition drastically reduced the HMS contrast relative to the noise region (Supplementary Figure S2B). Even under these challenging conditions, the detection algorithm effectively identified the robot down to a diameter of 1.6 mm, demonstrating the reliability and precision of our miniature robot imaging system.

We created the virtual reality environment using Unity®, which is a cross-platform game development engine and platform to create 2D and 3D virtual reality experiences. Besides Unity®, other platforms, including Gazebo, an open-source 3D robotics simulator, and CoppeliaSim, which is formerly known as Virtual Robot Experimentation Platform (V-REP) can offer their own unique features. For instance, Gazebo provides direct connectivity with the Robot Operating System (ROS) interface, which can be particularly beneficial for seamless integration with robotic hardware. We opted for Unity® due to its robust support for augmented and virtual reality applications (Melo et al., 2019). Unity® allows for seamless integration with other Unity®-based applications, simulations, and devices, which opens up significant opportunities to enhance our system’s performance and expand its capabilities in future three-dimensional control using single-source two-dimensional fluoroscopic image data.

The virtual reality environment contained a digital twin of the robot workspace and the HMS avatar. Then, we established a robust connection between the real-world dimensions and stream frame size with those in the virtual reality environment by developing a spatial calibration algorithm. This calibration process was conducted using the locations of two endpoints on our initial X-ray image of the real environment. If these endpoints are not distinctly visible due to their X-ray contrasts, fiducials may be placed to mark these points, as shown in Supplementary Figure S3A. We then registered these pixel position data of the fiducials on the image and recorded their pixel locations. The pixel-to-mm conversion was calculated by measuring the pixel distance between these two points relative to the real length of a known size object, such as the phantom we used as a model. Next, we positioned the digital twin at the first marked point and adjusted its orientation until it aligns precisely with the second marked point. This process ensures that the position and rotation of the real phantom are accurately mirrored in its digital twin, reducing the mismatch to several pixels with eye selection. To ensure accuracy of the relative initial positional information of the HMS within the real environment, the spatial calibration must be performed prior to each procedure.

To validate precise alignment between the avatar HMS position and its actual location, we conducted a tracking experiment by using an RGB camera and C-arm to record navigation videos (Figures 2C, Supplementary Figure S3B, Supplementary Movie S2). In processing the videos, the identifier data of HMS were captured for every frame and transmitted to Unity® using ROS2, which is facilitated by a custom Python 3 script tailored for this purpose. Using this method, we could continuously update the robot’s positional data in the HMS avatar within the digital twin as it moves in the physical world. With background calculations ongoing, the time delay between real-world and virtual-world data remained consistently below 100 ms, around 20–25 ms. This slight delay was largely negligible and undetectable by human robot operator. Unity®'s visual interface further allows the display of derived data, such as the instantaneous or time-average velocity.

### Virtual reality-enhanced real-time localization and steering control

**FIGURE 3:**
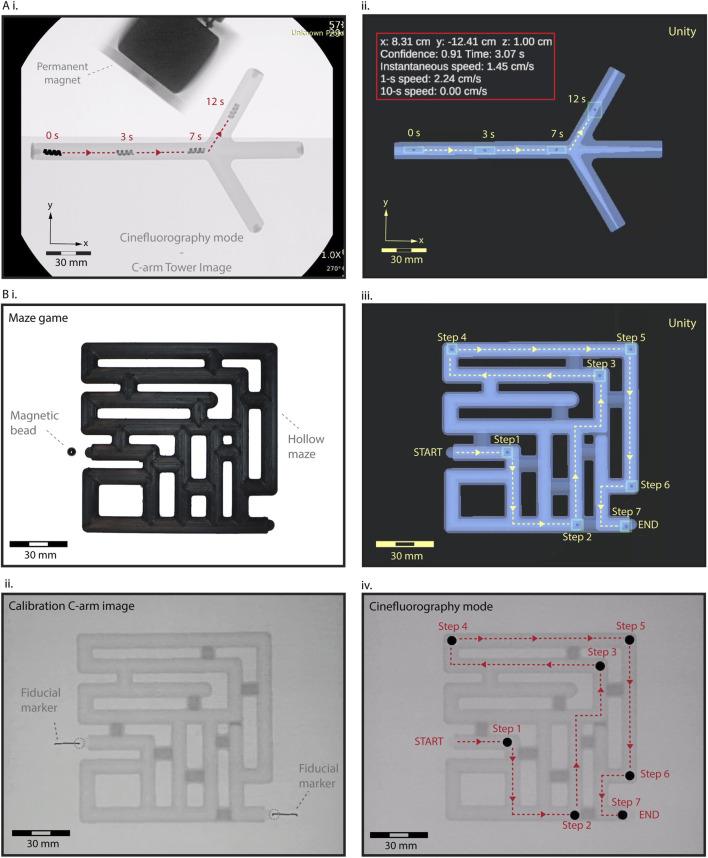
Fluoroscopic-guided real-time localization and telerobotic steering control in the digital twin interface. **(A)** (i) A real-time validation and control HMS steering. As the magnet and a segment of the robot arm traverse the imaging field, the relative contrast between the HMS and background visibility fluctuates. However, the detection algorithm is not affected by these fluctuations, maintaining the tracking robustness (ii) Spatiotemporally precise steering control of HMS from the virtual feedback within the digital twin. **(B)** A maze-solving game to demonstrate tracking and steering capabilities using the virtual feedback. (i) Initial RGB camera image showing a closed maze setup with a NdFeB magnet bead positioned at the start. (ii) The first X-ray image captures the maze with fiducial markers, used to calibrate and align the virtual and real-world coordinates before initiating telerobotic navigation. (iii) Sequential tracking and navigation of the bead through the virtual maze twin, from START to END, using stepwise control of the robot arm for remote magnetic actuation. (iv) Final validation of the magnetic bead’s localization within the maze under the C-arm, confirming the accuracy of the virtual navigation system.

Next, we demonstrated the feasibility of real-time steering of HMS under cine-mode fluoroscopic guidance (image acquisition rate ∼15 fps), augmented by virtual reality enhancement. We positioned a camera to capture the C-arm tower screen’s output, streaming this image data directly to our main computer (Supplementary Figure S4). The background algorithms for object detection, calibration, and localization seamlessly processed the live stream, transmitting identifier data to continuously update the HMS avatar within the digital twin with a rate of ∼15 fps. Tracking HMS took place with a short delay of approximately 7 ms in the virtual interface, allowing us to control the remote magnet’s position through feedback from the digital twin and precisely steer the robot into the side branch without overshooting the branching point (Figure 3A and Supplementary Movie S3). The detection algorithm maintained robust tracking as the magnet and a segment of the robot arm traverse the imaging field, causing fluctuations in the relative contrast between the HMS.

To further demonstrate the effectiveness of localization and steering capabilities using the virtual reality interface, we conducted a test using a 5 mm NdFeB sphere bead in a maze game. The fully enclosed maze featured a *START* and *END* point, along with multiple dead ends (Figure 3B). We generated the digital twin of the maze, calibrating its coordinate systems and dimensions with the physical maze using X-ray visible fiducials. We successfully navigated the bead through the maze in seven steps to the exit by instrumenting the magnetic fields based on the virtual feedback, demonstrating seamless integration of our virtual enhancement method to complement the fluoroscopic imaging in steering a miniature robot (Supplementary Movie S3).

## Discussion

### Envisioning virtual enhancement in a future miniature robot steering *in vivo*

**FIGURE 4:**
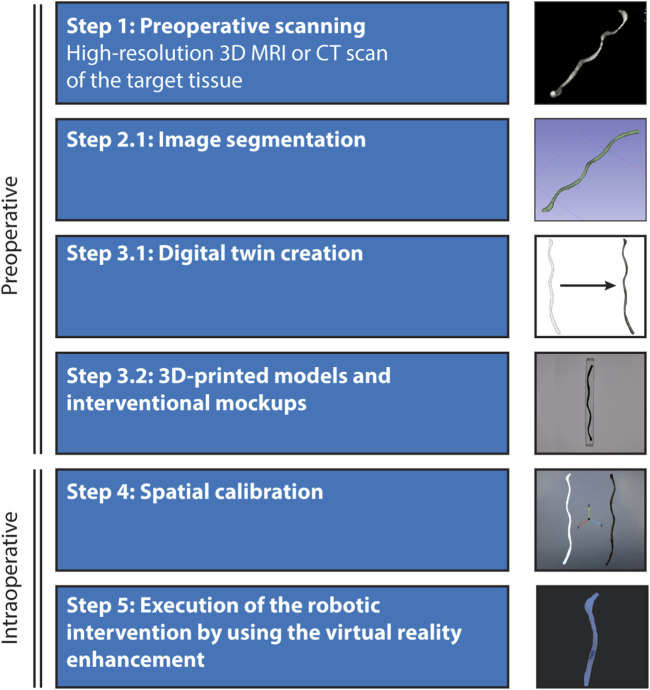
Envisioned process for the localization and steering of untethered miniature robots *in vivo*. Integrating virtual reality enhancement of the robot workspace to a live animal operation involves five main steps: *Preoperative scanning*- Initiation of the process with high-resolution scanning of the target tissue or organ using advanced 3D imaging systems such as MRI or CT. *Image segmentation and digital twin creation*- Detailed segmentation of the scanned images to construct a digital twin of the anatomical details within the virtual reality environment. This digital representation is crucial for accurate navigation and intervention planning. *3D-printed models and surgical mock-ups*- Utilization of segmented data to create 3D-printed physical models, providing tangible references for preoperative analysis and rehearsal. *Spatial calibration and execution*- Ensuring that the coordinate systems of the virtual and real environments are fully synchronized to facilitate the safe performance of the robotic interventions, supported by virtual tracking and localization feedback.

Our methodology holds significant promise for the translational development of miniature robot systems. To enhance visual feedback and user intuitiveness, we propose a pre-operative preparation phase where a digital twin of the target tissue is created. This involves constructing a detailed robotic workspace and anatomical model of the target animal or human tissues using high-resolution MRI or CT scans (Figure 4). These scans are then segmented to develop a digital twin that is seamlessly integrated into the virtual reality environment. Before the starting the intervention, spatial calibration and alignment are conducted to match the coordinates with the actual patient’s positioning and orientation, facilitated by the use of assistive fiducial markers. Additionally, during the preoperative stage, 3D-printed interventional mock-ups can be produced to trial the virtual reality environment, allowing for the meticulous planning and physical trials of the of robotic interventions in the lab before transitioning to the operating room for the animal or patient.

### Open challenges and future research

As a moving concept, digital twins are evolving beyond static virtual representations of human organs and tissues to become dynamic, model-driven interfaces that accurately reflect current conditions and predict future physiological events (Trevena et al., 2024). In this context, integrating medical miniature robots with digital twin platforms represents a strategic alliance in their translational development. This could include developing patient-specific physiological models that integrate various intraoperative sensory data, such as changes in body motion, blood flow rate, and vascular dynamics, into the digital twin model. For example, we plan to incorporate real-time flow data into our robotic control systems to enhance navigation accuracy under varying conditions. In phantom models, we will study this by integrating both in-flow data and ultrasound-based sensors to study responsive navigation strategies. For *in vivo* applications, our approach will involve using ultrasound to gather real-time flow data that will be directly integrated into our magnetic navigation algorithms. This will enable our system to dynamically adjust the robot’s kinematic and safety inputs based on real-time changes in flow conditions. By doing so, we aim to ensure that the robot’s actions are both adaptive and autonomous, enhancing navigation precision in environments that closely mimic clinical settings. Thus, virtual reality environments at the bedside are envisioned to consist of various anatomical and physiological modules used for both pre-procedure planning and intra-procedure simulation.

Although C-arm-guided procedures are commonplace in clinical settings, the exposure to ionizing radiation remains a significant concern. To the best of our knowledge, demonstrations of previous miniature robots moving under X-ray imaging have not reliably or reproducibly characterized their relative contrast density. Milli- and microrobots composed of materials that provide a higher total contrast than the clinical reference could potentially operate with a lower X-ray dose from the C-arm. This reduction could enhance safety for both patients and physicians by decreasing radiation exposure during procedures. To further reduce radiation risks, developing new prediction models is a viable approach. Such models would create on and off times for X-ray. They allow for the continuous operation and precise guidance of the robot even when the X-ray system is inactive by predicting the robot’s position. Reactivating the X-ray system at specific intervals would enable the integration of new data to refine these predictions, thus improving the accuracy of the localization and steering. These models could be enabler in procedures involving miniature robots, where consistent tracking is essential, but prolonged radiation exposure can be harmful.

While these opportunities have the potential for significant impact on patient-specific innovative treatment methods, several technical challenges must be addressed head-on in developing a clinically useful multi-functional virtual reality environments for miniature robot control. To safely localize, track and navigate a miniature robot *in vivo*, a primary challenge is the fluoroscopic-guided control in 3D using 2D image data from a single x-ray source. Another challenge is ensuring accurate position, orientation and scale matching of the target anatomical unit to its digital twin. To enhance positional referencing, we aim to improve our object detection mechanism by expanding its capability to detect X-ray fiducial markers, as well. This functionality will enable automatic detection of fiducials systematically placed around the target tissue, allowing us to define its shape more accurately and repeatedly. Such fiducial-guided interventions are a staple in precision therapies for accurate spatial registration (O’Neill et al., 2016). In 2D images, minimum three fiducial markers are needed to determine the position, orientation and scale of the object, while in 3D, minimum four fiducials will be needed. This process will dynamically update any small changes in tissue’s position and orientation, reflecting any major disturbances in the digital twin for real-time control.

Our ultimate goal with this research direction is to develop a fully autonomous miniature robot magnetic navigation system under the dynamic feedback of virtual interface, integrating comprehensive sensor data related to physiology and anatomy into a central computer framework. This cohesive control mechanism, bolstered by superior data processing capabilities of autonomous systems compared to human operators, can adaptively mitigate disturbances such as physiological changes and patient movement, thereby reducing human intervention and enhancing procedural safety. Our future research will focus on incorporating specific methods for accurate three-dimensional path planning, advanced prediction algorithms, and magnetic control strategies for endovascular and neurointerventional applications.

## Conclusion

This proof-of-concept study has successfully demonstrated real-time fluoroscopic detection, tracking, and steering of untethered miniature robots within a static, two-dimensional phantom testbed by using virtual reality enhancement. The feasibility of this methodology lays a promising foundation for the development of sophisticated three-dimensional control capabilities for the fluoroscopic guided robotic navigation within animals and the human body. By integrating rich, real-time physiological and advanced predictive models for imaging and magnetic navigation, we can significantly enhance the control and dexterity of miniature robots while reducing dependence on continuous radiation exposure. The integration of virtual reality enhancement can become a crucial enabler for meeting the rigorous safety and efficacy standards required for emerging miniature robot technologies in realistic clinical scenarios.

## Materials and methods

### Materials

Unless described otherwise in its relevant context, all chemicals were purchased and used from Sigma Aldrich in high purity.

### Magnetic field control

An LBR Med 7 R800 robot arm (KUKA) with seven degrees of freedom was employed to control the position and orientation control of the global magnetic field in the workspace of the millirobot. Our KUKA robot arm’s software version is V1.5.4-2 and KUKA Robot arm programmed with its unique application (KUKA Sunrise Workbench – 2.6.5_6). Attached to the robot arm end effector, a custom-designed DC motor (Maxon)-run part facilitates the high-performance rotation of an external permanent magnet (K&J Magnetics) up to ∼100 Hz.

### X-ray fluoroscopy imaging

X-ray images were captured in cinefluoroscopic and digital subtraction angiographic modes using an OEC 3D C-arm (General Electric, Boston, MA). The DICOM image files from the C-arm were converted to.JPEG format and.MP4 using RadiAnt DICOM Viewer (version 2023.1, 64-bit) software.

### Camera imaging

Video recordings in Figure 2 were captured using a Blackfly S Sony IMX546 2/3″CMOS camera. For real-time magnetic navigation system with the C-arm described in Figure 3, a Logitech webcam camera (Logitech Brio 4K webcam V-U0040), with 640 × 480 frame size, was localized in front of the C-arm tower monitor to capture and transfer the image data to Unity® (Supplemetnary Figure S4B).

### Fabrication of HMS

The fabrication of HMS involved designing a master positive mold and a pin with predefined dimensions using Fusion 360 (Autodesk, San Francisco, California), followed by their 3D printing using a commercial stereolithography (SLA) printer (FormLabs 3B+, FormLabs, Somerville, MA). Subsequently, the master positive mold and pin were utilized to create a negative mold made from silicon elastomer. A mixture with a 10:1 mass ratio of SYLGARD™ 184 Silicone Elastomer Base and Curing Agent (DOW Chemical Company) was prepared and cured in a heater at 80°C for 2 h. Prior to the subsequent step, the surface of the negative mold was passivated using plasma treatment and alcohol passivation, as described previously (Kim et al., 2019). The silicon elastomer: NdFeB powder from Magnequench (D50 = 5 μm) mass ratio was 1:4. The thoroughly mixed precursor composite was then degassed under vacuum and subsequently cast into the passivated negative PDMS mold and cured in the heater at 80°C for 2 h. Following the curing, the helical swimmer was gently demolded. Finally, the HMS was magnetized in a direction perpendicular to its major helical axis using our custom-made magnetic yoke under 1.1 T. The magnetization protocol is described in detail in Supplementary Figure S1.

### Real-world models

Phantom models and their assistive parts with pre-defined dimensions were designed using Fusion 360. The transparent phantom with fluid-filled closed channel in Figures 1–3 was designed with a length of 186 mm and a width of 12 mm. To illustrate the navigation capabilities of HMS through branches of varying sizes, a phantom with four channels was meticulously constructed. The initial channel maintains a consistent diameter of 6 mm, while the remaining three channels adopt a conical structure, commencing with diameters of 6 mm and gradually narrowing to 1 mm, 2 mm, and 3 mm, respectively. A sacrificial mold was 3D printed using a K1 Mx AI fast 3D printer (Creality, Shenzhen, China) using acrylonitrile butadiene styrene (ABS) filament (Creality). This mold is then sprayed with non-adhesive ease release 200 spray (Mann release technologies, Macungie, PA, United States) and placed in a container. Then, the container was filled with SYLGARD™ 184 Silicone Elastomer Kit, where components A and B were mixed in a 1:10 ratio. Following overnight curing at room temperature, the sacrificial ABS mold was dissolved with acetone the next day, resulting in the phantom in its final form (Supplementary Figure S5). The maze was 3D printed from the ABS filament, fully closed and opaque, to demonstrate real-time navigation control in the virtual interface.

### Digital twins

The digital twins of the phantom testbeds were created by importing the.OBJ file of the phantom design (Fusion 360) into the Unity® 2022.3.0f1 editor version (Unity® Technologies).

### Spatial calibration

To achieve spatial alignment of the phantom testbeds and digital twins, a calibration method was developed. Prior to the navigation task, an initial image was captured to (i) match the frame size of the input video to the virtual frame size and correct (ii) position and (iii) rotation of the object within the frame to the digital twin. X-ray-opaque fiducials were used to identify the starting and ending positions of the phantoms defined on the image, followed by pixel-to-mm conversion. To execute this task, a function was added to the Python and C# codes. This function uses the initial image to register the fiducials. The first fiducial was used to match starting points of real phantom and digital twin and second was used to match their rotation around the starting point. Subsequently, Unity® positions the digital twin of the phantom by adjusting its position and angle to match accurately the real-world positioning.

### Communication network

A new version of the Robot Operating system, ROS2, more specifically ROS2 Humble, was used to create a communication network for all separate system works within our project. Everything works within our local network and each system can send and retrieve data by publishing and subscribing to the related topics, data packages, on ROS2 network. In our work, we have python codes for object detection and ROS2 communication, and C# codes for Unity® connection and virtual reality environment control. For future uses, everything can be connected to the internet network for untethered communication between each system which can lead cross-continental connection. After the spatial calibration, the connection of the Unity® system with the ROS2 network was established using ROS-TCP-Endpoint libraries from Unity® Technologies. With this connection, detected HMS data could be received, and its digital twin position could be adjusted in real time. As shown in Figure 2B, their positions match accurately without any detectable mismatching in the given pixel size.

### Robot detection model

A dataset consisting of 318 images of HMS was created from the videos of robot navigation within the phantoms, captured by an RGB camera or C-arm, to train the object detection model. Roboflow platform (Roboflow, Inc., Des Moines, Iowa, United States) was used to create and annotate the database (Dwyer et al., 2024). The built-in data augmentation functions in Roboflow were also utilized to expand the robot image dataset for training, creating new iterated images of the originals with various defects and changes such as flip, rotation, blur, saturation, brightness, exposure, and grayscale. To facilitate testing of our model’s success, validation and test sets were needed; thus, this expansion was applied after separating some images for these sets to preserve the original form of our detection target. Consequently, a total of 1,210 images were obtained, with 92% (1,115 images) in the *Train set*, 5% (63 images) in the *Validation set*, and 3% (32 images) in the *Test set*. Following dataset preparation, a new version of the You Only Look Once (YOLOv8, Ultralytics, MD, United States) algorithm was employed for object detection (Jocher et al., 2023). The model underwent training for 300 iterations to reduce object losses and missed detections. Upon completion of training, a weight file containing all the learned detections from the training was generated and made ready for use in our system. Subsequently, a Python script was developed to utilize the trained dataset for robot detection on pre-recorded videos or real-time footage. In the recreation of the twin HMS in Unity®, a detected robot within the imaging frame was outlined with a bounding box, and its center point was marked with a circle in the background. The position of an HMS detected with over 90% confidence value was sent to Unity® over the ROS2 network. For the visual validation of a detected HMS, a bounding box was also printed in the recorded videos around HMS, as shown in Figure 2, Supplementary Figures S2, S3, whenever a detection event occurred.

## Data Availability

The original contributions presented in the study are included in the article/Supplementary Material, further inquiries can be directed to the corresponding author.
