# Supplementary material for: X-ray fluoroscopy guided localization and steering of miniature robots using virtual reality enhancement
Source: Front Robot AI. 2024 Nov 13;11:1495445. doi: 10.3389/frobt.2024.1495445 (PMC11599259; doi:10.3389/frobt.2024.1495445)
Supplement: Supplementary file 2 [file Presentation1.pdf]

Supplementary Information File

for

X-ray Fluoroscopy Guided Localization and Steering of Miniature Robots Using Virtual Reality Enhancement

Husnu Halid Alabay, Tuan-Anh Le, Hakan Ceylan

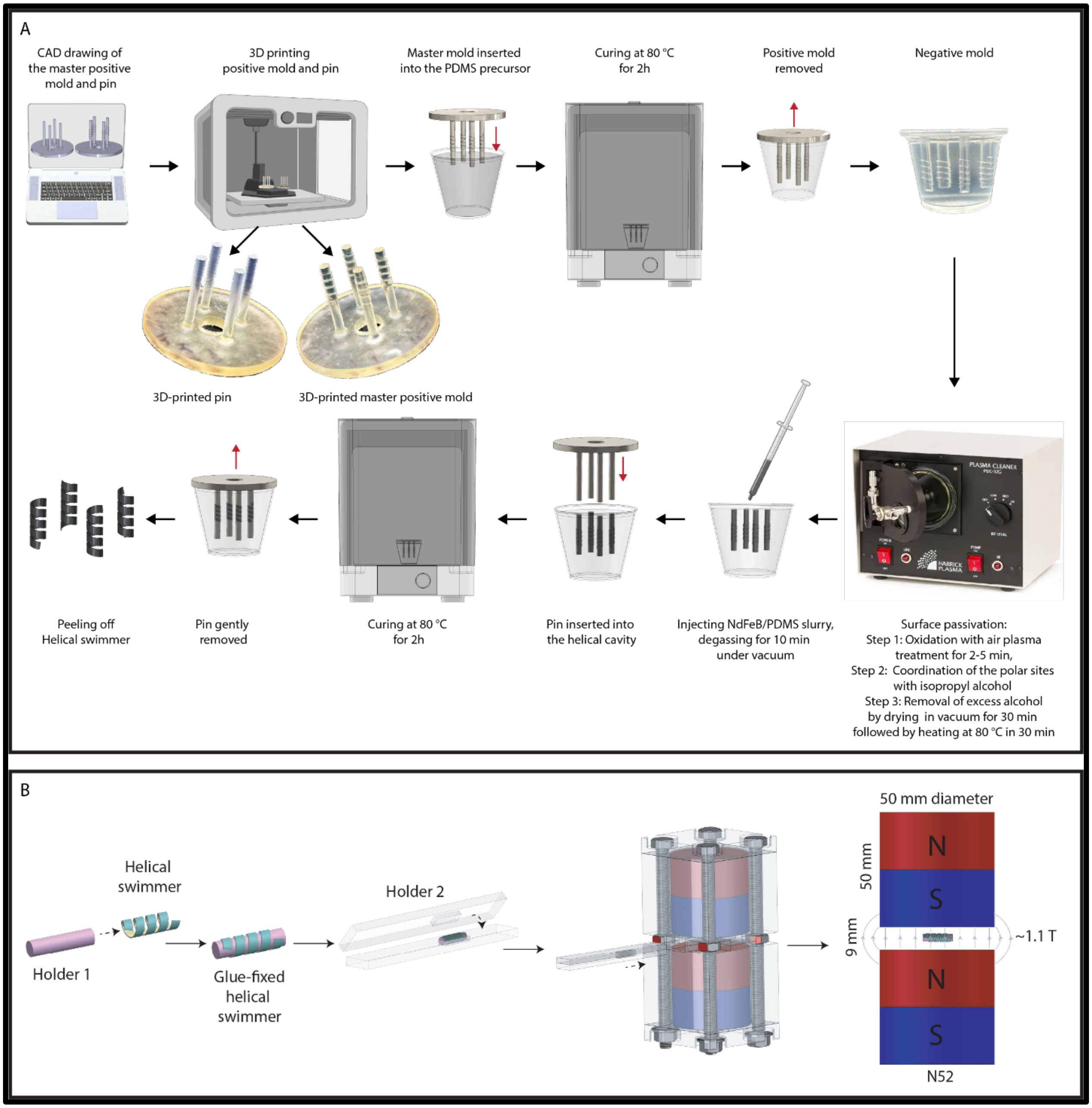

Figure S1. (A) Fabrication methods and production steps HMS. (B) Magnetization of HMS.

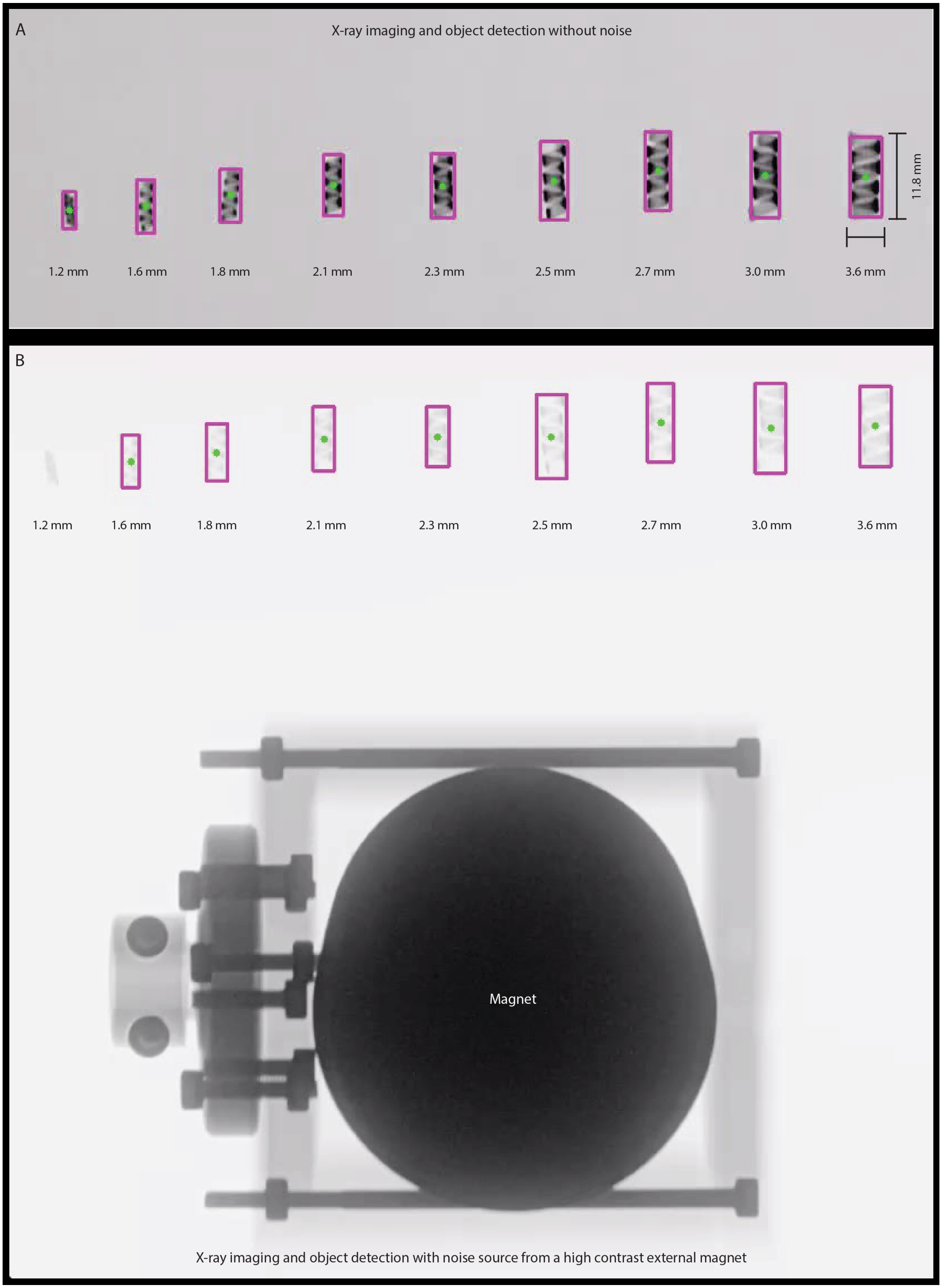
A
X-ray imaging and object detection without noise
11.8 mm
1.2 mm
1.6 mm
1.8 mm
2.1 mm
2.3 mm
2.5 mm
2.7 mm
3.0 mm
3.6 mm
B
1.2 mm
1.6 mm
1.8 mm
2.1 mm
2.3 mm
2.5 mm
2.7 mm
3.0 mm
3.6 mm
Magnet
X-ray imaging and object detection with noise source from a high contrast external magnet

Figure S2. Detection and imaging Range of HMS across the robot diameters from 1.2 mm to 3.6 mm. (A) Successful fluoroscopic visibility and detection of HMS within this size range under X-ray fluoroscopy in a clear field, free from external interference. (B) Detection of HMS as small as 1.6 mm diameter, demonstrating the robustness of the robot detection, despite the extreme distortion caused by the higher contrast of a permanent cylindrical magnet. Our detection algorithm is also trained to selectively identify HMS and distinguish it from screws within the image space that secure the magnet encapsulation system.

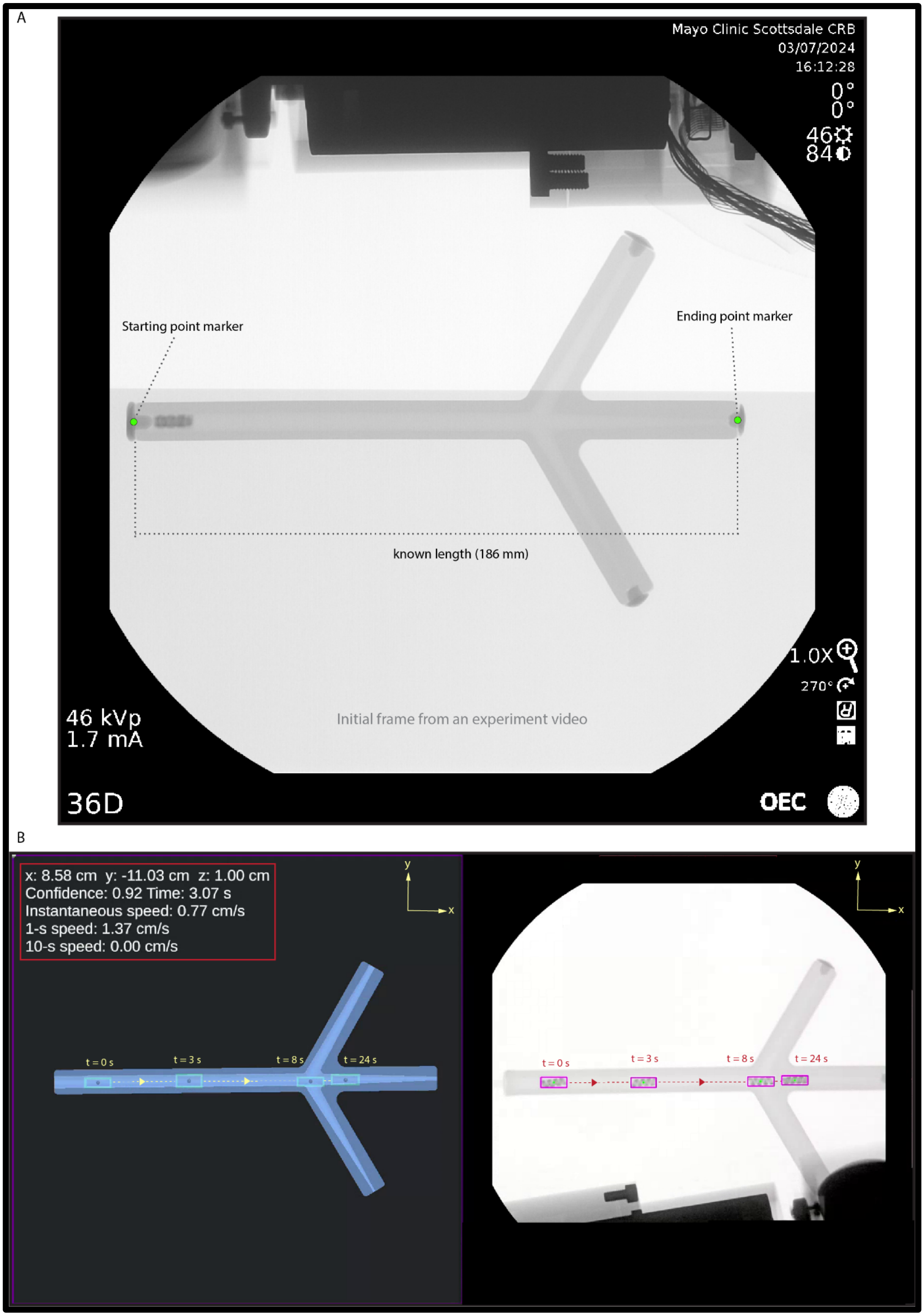
A
Mayo Clinic Scottsdale CRB
03/07/2024
16:12:28
Starting point marker
Ending point marker
known length (186 mm)
Initial frame from an experiment video
46 kVp
1.7 mA
36D
1.0X
270°
OEC
B
x: 8.58 cm y: -11.03 cm z: 1.00 cm
Confidence: 0.92 Time: 3.07 s
Instantaneous speed: 0.77 cm/s
1-s speed: 1.37 cm/s
10-s speed: 0.00 cm/s
t = 0 s
t = 3 s
t = 8 s
t = 24 s
y
x

Figure S3. (A) Spatial calibration uses green circles to define start and end position of the phantom and align it with the virtual twin for accurate overlay. (B) Accurate spatial and temporal synchronization of an HMS navigation validated in a C-arm cinefluography video in the virtual environments with time stamps, position, and HMS speed data.

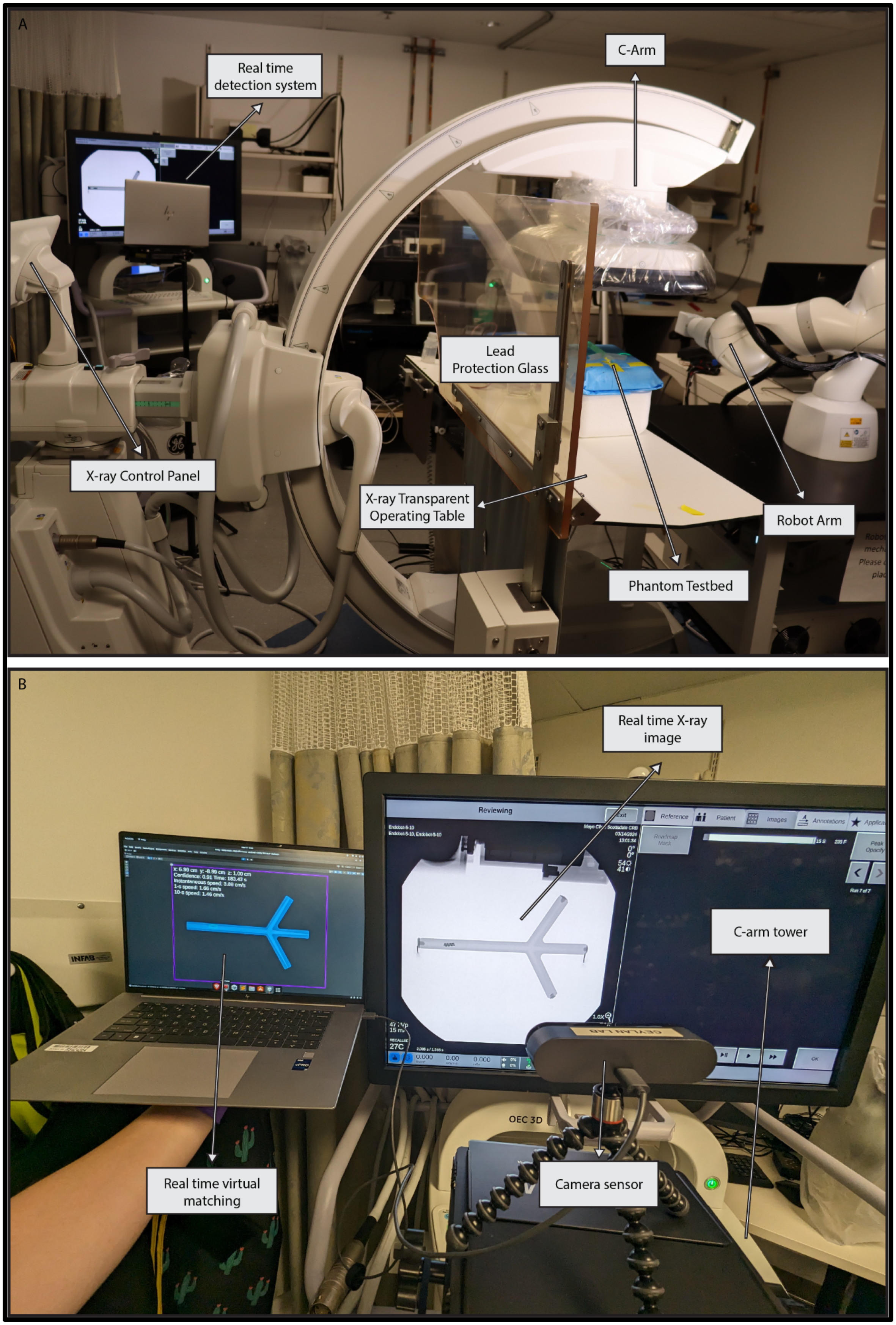
A
Real time detection system
C-Arm
Lead Protection Glass
X-ray Control Panel
X-ray Transparent Operating Table
Robot Arm
Phantom Testbed
B
Real time X-ray image
Reviewing
C-arm tower
OEC 3D
Real time virtual matching
Camera sensor

Figure S4. (A) Experimental setup for real-time detection, localization, and navigation of HMS from the C-arm image tower. (B) Close-up view showing real-time integration of the X-ray image, camera sensor, and virtual matching. In the development process, we occasionally used a computer web camera or a separate camera (Logitech) to capture images from the C-arm.

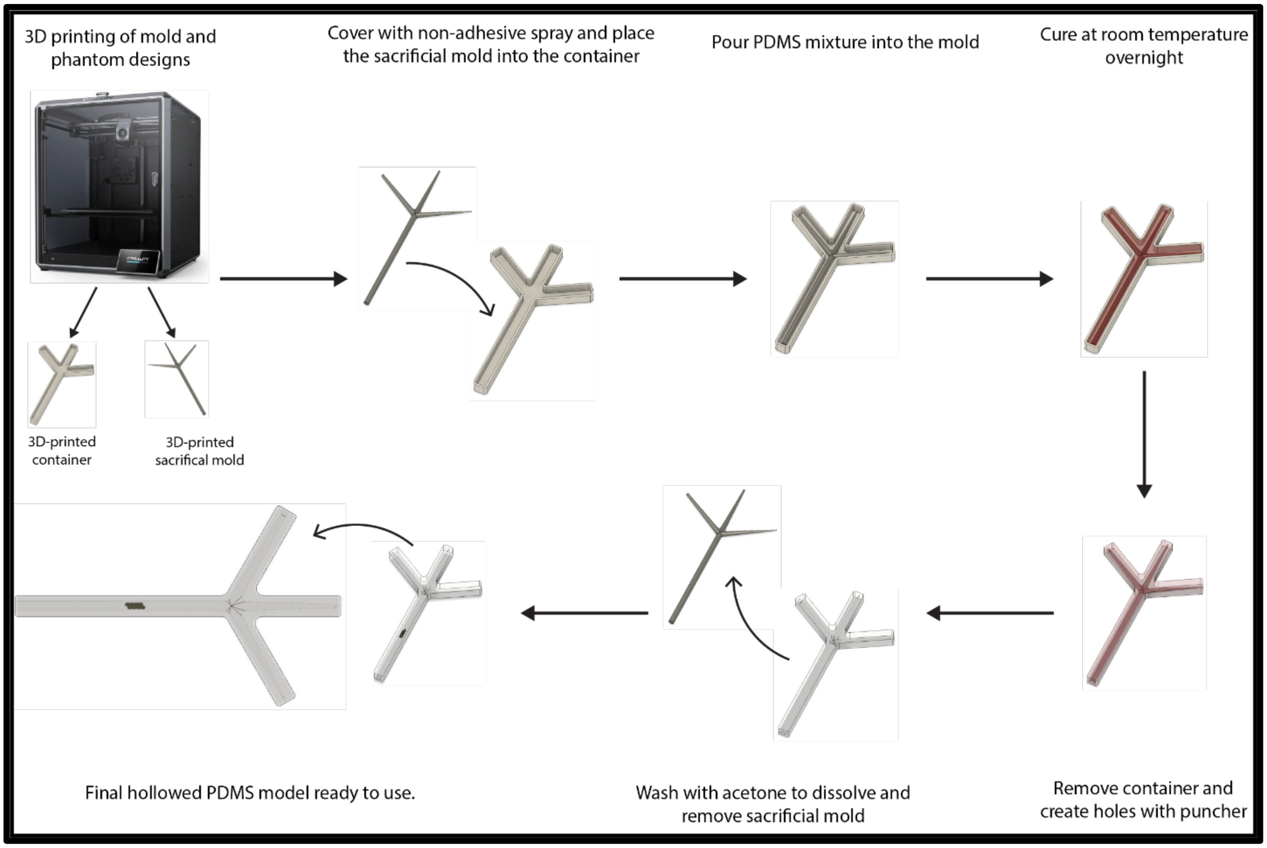

Figure S5. Fabrication of the two-dimensional phantom testbed.
